# Pentamethinium Salts Nanocomposite for Electrochemical Detection of Heparin

**DOI:** 10.3390/ma14185357

**Published:** 2021-09-17

**Authors:** Tatiana V. Shishkanova, Tomáš Bříza, Pavel Řezanka, Zdeněk Kejík, Milan Jakubek

**Affiliations:** 1 Department of Analytical Chemistry, University of Chemistry and Technology, Prague, Technická 5, 166 28 Prague 6, Czech Republic; pavel.rezanka@vscht.cz (P.Ř.); milan.jakubek@vscht.cz (M.J.); 2 BIOCEV, First Faculty of Medicine, Charles University, Průmyslová 595, 252 50 Vestec, Czech Republic; tomas.briza@lf1.cuni.cz (T.B.); zdenek.kejik@lf1.cuni.cz (Z.K.); 3 Department of Paediatrics and Inherited Metabolic Disorders, First Faculty of Medicine, Charles University and General University Hospital, Kateřinská 1660/32, 121 08 Prague 2, Czech Republic

**Keywords:** gold nanoparticles, γ-substituted pentamethinium salts, nanocomposite, voltammetric sensor, heparin

## Abstract

This study presents a simple route to heparin detection and develops a voltammetric approach using supramolecular principles and nanomaterials. Nanocomposites, including gold nanoparticles (AuNPs) and γ-substituted pentamethinium salts (PMS) deposited on a glass carbon (GC) electrode surface (GC/AuNPs/PMS) and covered by a plasticized poly(vinyl chloride) (PVC) membrane, are proposed for heparin detection. The conductivity of the nonconducting PVC-plasticized membrane is guaranteed by AuNPs, and the selectivity is provided by the interaction between γ-substituted PMS and anionic analytes. In order to extend the linear range, it is necessary to apply a solvent compatible with PVC-plasticized membrane, namely tetrahydrofuran. The proposed voltammetric sensor showed a concentration dependence from 1.72 up to 45.02 IU mL^−1^ heparin and was used for heparin detection in saline and biological samples with recovery of 95.1–100.9%.

## 1. Introduction

The contact between the recognizing site immobilized on the membrane and the electrode surface presents a challenge in field sensors. Potentiometric sensors with solid-supported membranes provide such an example. To stabilize the potentiometric signal, a hydrophobic conducting polymer such as poly(3-octylthiophene) is placed between the membrane and metal. Amemiya et al. demonstrated that the solid-supported ion-selective electrode can be successfully applied as a voltammetric sensor for detecting biomedically important polyion such as heparin [1]. However, it should be kept in mind that the conducting polymer is a polymer with redox properties and can be affected by both the environment and other redox active components. This problem can be solved by introducing novel nanocomposite materials. Nanocomposites may be materials composed of polymers and nanomaterials. Nanomaterials have been extensively exploited for signal amplification, the improvement of detection efficiency, and as redox mediators in electrochemical sensors [2,3,4,5,6,7]. Recently, nanocomposites based on polyaniline, polypyrrole with entrapped carbon nanotubes (CNTs), single-walled (SWNTs) and multiwalled nanotubes (MWNTs) have received significant popularity due to their ease in preparation through copolymerization by chemical or electrochemical approaches [8,9].

**Scheme 1:**
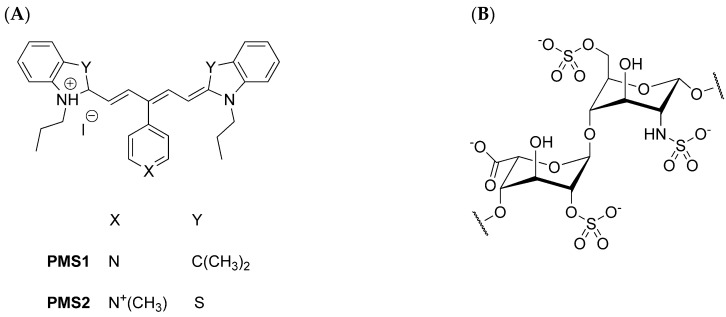
γ-substituted pentamethinium salts proposed for heparin binding (**A**) and the structure of the major disaccharide unit of heparin (**B**).

Herein, we propose a novel nanocomposite: a mixture of AuNPs and supramolecular receptors dispersed in an organic solvent (methanol or tetrahydrofuran), that is physically adsorbed onto the electrode surface and covered by a nonconducting polymeric matrix to prevent the leaching of the active component. γ-substituted pentamethinium salts, (PMS) differing in lipophilicity, were used as receptors. Polymethine (cyanine) dyes are widely used as probes for biomacromolecules [10,11]. This is due to a flexible polymethine chain in their molecules and the ability to form noncovalent complexes with biomacromolecules. One of such dyes, 3,3′,9-trimethylthiacarbocyanine iodide (Cyan 2), was studied as a spectral probe for interaction with hyaluronic acid (HA) [11] that represents an important class of biological macromolecules—glycosaminoglycans [12,13,14,15]. Therefore, it was of interest to investigate the interaction between polymethine (cyanine) dyes and other glycosaminoglycans, such as heparin. Heparin is used as an anticoagulant drug able to accelerate the rate at which antithrombin inhibits serine proteases in the blood coagulation cascade [16,17]. Recently, various electrochemical sensors have been reported to detect heparin using an ion-exchanger mechanism [18]. In this paper, we report the detection of heparin using voltammetric sensors based on pentamethinium salts’ nanocomposite. The low cost and ease of operation of such sensors should make them ideal tools for pharmaceutical applications. The structure framework of γ-substituted PMS (Scheme 1) includes cationic charge for coulombic interaction and hydrophobic units for increasing lipophilicity. Thus, γ-substituted PMSs are assumed to have good compatibility with the PVC matrix and good response towards anionic species. The mixture consisting of AuNPs and γ-substituted PMS was inserted onto a glassy carbon (GC) electrode surface and then coated by a thin layer of NPOE-plasticized PVC. This electrode was applied to detect heparin.

## 2. Materials and Methods

### 2.1. Reagents and Instrumentation

The following chemicals were used for the experiments: γ-substituted pentamethinium salts (PMS) (Scheme 1), which were synthesized as described previously in [19,20,21], potassium hexacyanoferrate(II) and hexacyanoferrate(III) (Lachema, Neratovice, Czech Republic), potassium chloride (Eutech, Nijkerk, Netherlands), unfractionated heparin sodium (177.0 IU mg^−1^, SPOFA, Prague, Czech Republic), 2-nitrophenyl octyl ether (Selectophore, Fluka, Buchs, Switzerland), polyvinylchloride (PVC, high molecular weight, selectophore, Fluka, Buchs, Switzerland), tetrahydrofuran (THF, Fluka, Buchs, Switzerland) and methanol (Fluka, Buchs, Switzerland). All chemicals were of analytical reagent grade and used as received without any further purification. The mixture of 1.0 mmol L^−1^ K_3_[Fe(CN)_6_] and 1.0 mmol L^−1^ K_4_[Fe(CN)_6_] (1:1) was prepared in 0.1 mol L^−1^ phosphate buffer with pH = 7.0 and 0.14 mol L^−1^ NaCl (PBS) was added. A 1.95 mg mL^−1^ stock solution of heparin was prepared by dissolving 0.0078 g of heparin sodium reagent (177.0 IU mg^−1^, SPOFA, Prague, Czech Republic) in 0.1 mol L^−1^ PBS and diluting this to 4 mL. The heparin stock solution was stored in the refrigerator at 6 ± 2 °C. Gold nanoparticles (AuNPs) were prepared as previously described [22]. Shortly after, 1 mL of 1% aqueous solution of potassium tetrachloroaurate(III) and 2.5 mL of 1% aqueous solution of trisodium citrate dihydrate were added to 100 mL of boiling water (under reflux). Boiling was continued for 10 min. During that time, the solution color changed from pale yellow to gray-blue, then to purple, and finally to wine-red. The reaction vessel was then allowed to cool to room temperature. The human serum samples diluted with 0.1 mol L^−1^ PBS (dilution ratio: 1:5) were obtained from the Department of Children and Adolescents, Faculty Hospital Královské Vinohrady (Prague).

Voltammetric measurements were carried out using a potentiostat/galvanostat Autolab pGST-12 (Metrohm Autolab B.V., Ultrecht, The Netherlands). A conventional three-electrode arrangement was used. The working electrode was a Glassy Carbon (GC) electrode (Elektrochemické Detektory s.r.o., Turnov, Czech Republic). The reference electrode was Ag/AgCl (3 mol L^−1^ KCl) (Elektrochemické Detektory s.r.o., Turnov, Czech Republic). The counter electrode was a Pt foil. UV–Vis spectra were recorded using a Varian Cary 400 SCAN UV-Vis spectrophotometer (BMI Surplus, Inc., Hanover, MA, USA). Solvent spectra were subtracted from all spectra. Data were collected from 200 nm to 800 nm with 1 nm resolution. Fluorescence spectra were recorded using a FluoroMate FS-2 fluorescence spectrometer (SCINCO, Seoul, Korea). Emission spectra were recorded at 590 nm excitation wavelength from 620 to 800 nm with 0.1 nm resolution.

### 2.2. Preparation of Pentamethinium Salts Nanocomposite

Pentamethinium salt (PMS1 or PMS2) (0.4 mg) was added to the solution of freshly prepared AuNPs (4 mL). After 2 days, unbound PMS species were removed by centrifugation of 2 mL followed by redispersion of the modified AuNPs in methanol. Another 2 mL was centrifugated, followed by redispersion of the modified AuNPs in THF. Centrifugation and redispersion in the correspondent solvents were repeated twice for formation AuNPs/PMS.

### 2.3. Electrode Modification

**Scheme 2:**
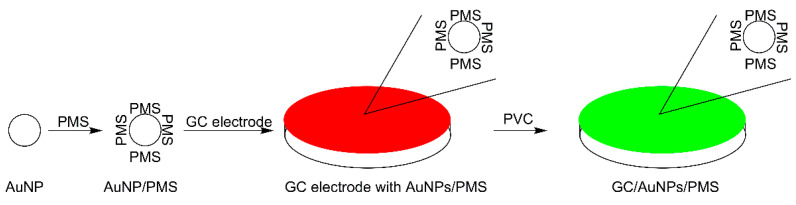
Process of GC electrode surface modification.

A priori, the surface of GC electrode was primarily cleaned mechanically using filter paper and then treated electrochemically by cycling the potential from −0.3 to 1.8 V in 0.5 mol L^−1^ H_2_SO_4_. Then, 20 μL of AuNPs/PMS dispersion prepared either in methanol or THF was deposited on a GC electrode surface. After the evaporation of organic solvent from the GC electrode surface, a PVC membrane mixture was drop-cast from a solution containing PVC: o-NPOE (66:34 wt %, *m* = 0.1 g) in 0.5 mL of THF. A total of 50 μL of the THF solution was drop-cast onto the modified GC electrode surface in the form of 10 μL aliquots, added every 5 min. The schematic illustration of the details concerning of the GC electrode surface modification is presented in Scheme 2. The micrographs of the layer of NPOE-plasticized membrane coating the electrode surface were taken with a scanning electron microscope, JSM 6400 (JEOL, Japan) (Supplementary Materials, Figure S1).

### 2.4. Electrode Characterization and Electrochemical Binding Studies

The voltammetric measurements were performed on unmodified(GC/AuNPs) and modified (GC/AuNPs/PMS) electrodes coated by PVC-plasticized membrane with the supporting electrolyte 1.0 mmol L^−1^ K_3_[Fe(CN)_6_] and 1.0 mmol L^−1^ K_4_[Fe(CN)_6_] (1:1) in 0.1 mol L^−1^ PBS at pH = 7.0. The signal was measured in the absence and presence of the analyte in question over the potential range of −0.5 to +1.0 V and −0.8 to +1.2 V, respectively, at a scan rate of 50 mV s^−^^1^. To eliminate electrode-to-electrode variation in the background signal, the following equation was applied:

*Signal* = (*I − I*_0_)/(*I*_0_ × 100%)

where *I*_0_ and *I* were the current response of the tested GC electrodes recorded in the supporting electrolyte before and after adding the different concentrations of the tested analyte. The heparin determination in the model samples was carried out using the standard addition method.

**Figure 1:**
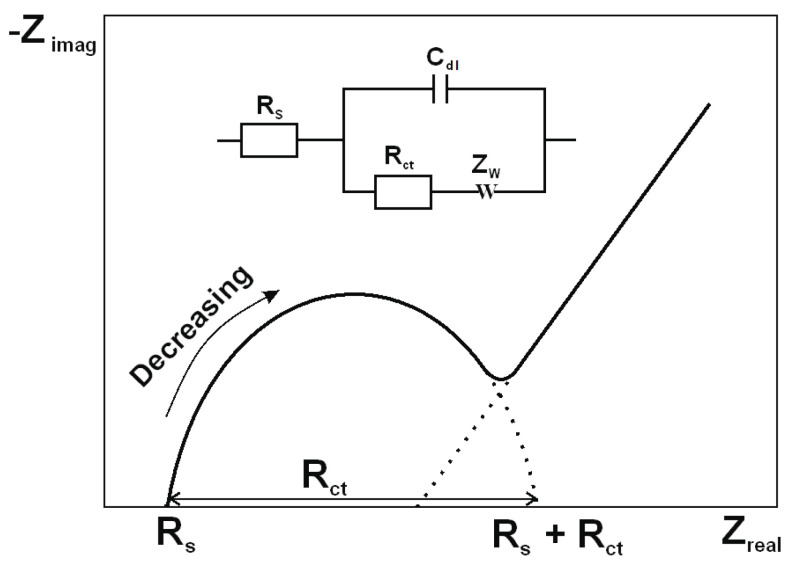
Nyquist plot resulting from electron impedance spectroscopy and Randles circuit was used for its fitting [23]. Symbols: *R*_s_—series resistance of solution, *C*_dl_—capacity of the double layer formed on the electrode surface, *R*_ct_—charge-transfer resistance between electrode and solution, *Z*_w_—Warburg impedance describing the diffusional resistance element.

The charge-transfer resistance of the GC/AuNPs/PMS-modified electrode surfaces was measured by an electrochemical impedance spectroscopy (EIS) technique in the supporting electrolyte with and without the analytes of interest using an Autolab PGSTAT-12 potentiostat/galvanostat supplied with FRA 2 modules for impedance measurements (Metrohm Autolab B.V., Ultrecht, The Netherlands). The three-electrode electrochemical cell consisted of a GC/AuNPs/PMS electrode as the working electrode, saturated Ag/AgCl as the reference electrode, and a platinum plate as the counter electrode. The EIS spectra were collected at a potential of 0.0 V in the frequency range of 100 kHz to 100 mHz (50 points), with the amplitude of the applied sine-wave potential being 10 mV. The Randles circuit (Figure 1) was used for the curve fitting of impedance spectra in the software package Nova 11 Autolab. 

## 3. Results

Under physiological conditions, heparin is a highly negatively charged glycosaminoglycan due to completely ionized carboxyl (COO^−^) and sulfate groups (OSO_3_^−^ and NHSO_3_^−^). The heparin chain exists in an extended helical conformation, primarily because of the high charge density. Although tightly coiled, heparin is not rigid, and it exploits this conformational flexibility in the interaction with the proteins responsible for heparin’s biological activity [24,25,26]. Cationic pentamethinium salts represent promising receptor systems for the interaction with negatively charged heparin [19,21]. Preliminary studies confirmed the affinity of PMS1 and PMS2 for heparin with log(*K*) equaled to 10.8 and 11.2, respectively, and the level of interference of the selected analytes [21]. It was shown that β-glucan, hyaluronic acid and alginic acid caused the least interference. In contrast to the PMS2 salt, the PMS1 salt showed a lower affinity for sulfated polysaccharides with a higher number of anionic groups. It was expected that increasing heparin concentration would lead to its selective interaction with pentamethinium salt, the displacement of iodide from the membrane layer, iodide release, and accumulation of negative charge on the phase boundary between the PVC-plasticized membrane and solution. As a result, redox markers were repulsed from the electrode surface and voltammetric signals decreased. This phenomenon allows for the observation of the dependency between the changes of voltammetric signal and the analyte concentration.

### 3.1. Characterization of Nanocomposites

**Figure 2:**
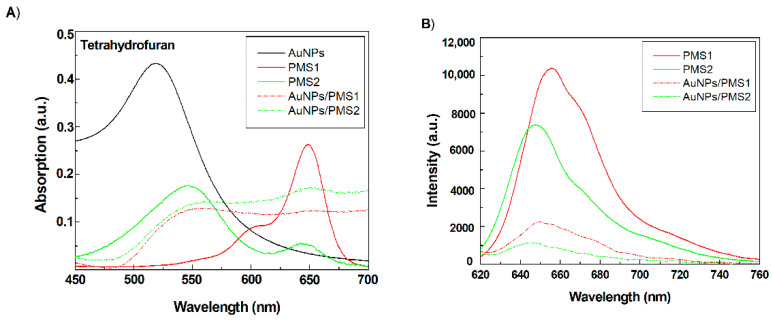
UV–Vis (**A**) and fluorescence (*λ*_ex_ = 590 nm, (**B**) spectra of a free (PMS, 1 μg mL^−1^) and immobilized (AuNPs/PMS) receptor recorded in tetrahydrofuran. For comparison, there is UV–Vis spectrum of the freshly prepared AuNPs. The fluorescence spectrum of AuNPs showed no signal.

The successful immobilization of PMS on to the surface of AuNPs was confirmed using UV–Vis and fluorescence spectroscopy. After the addition of PMSs to AuNPs solution, a shift was observed of the AuNPs peak that corresponds to the plasmon resonance from 521 nm (black curve) to 564 nm for both AuNPs/PMS1 (red curves) and AuNPs/PMS2 (green curves) (Figure 2A). This effect can be explained by the increasing size of AuNPs due to the anchoring of PMS on their surface [27]. Moreover, the peak characteristic for PMSs were clearly visible in the UV–Vis (λ = 650 nm, Figure 2A) and fluorescence spectra of purified AuNPs/PMS dispersion (Figure 2B). The formation of the nanocomposite is probably based on the combination of coulombic and π–π interactions as was previously described [27]. The UV–Vis and fluorescence spectra measured in MeOH were similar and therefore not shown.

### 3.2. Electrochemical Characterization of the Modified Electrode Surface

**Figure 3:**
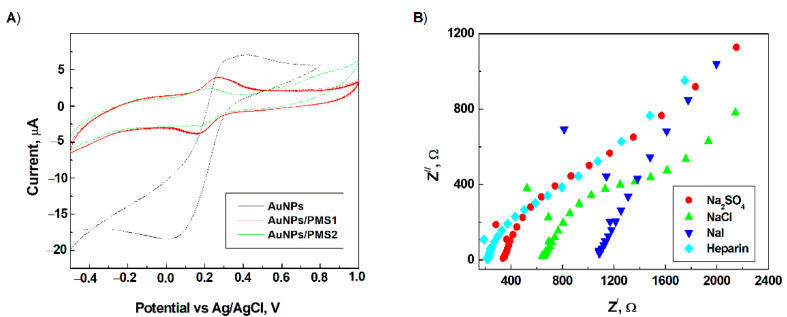
(**A**) Cyclic voltammetric response for the GC/AuNPs/PMS-modified electrodes (scan rate: 50 mV s^−1^). (**B**) The EIS spectra recorded for the GC/AuNPs/PMS1-modified electrode towards mono- (NaCl, NaI), di- (Na_2_SO_4_) and multi- (heparin) charged anionic species. Supporting electrolyte: 1 mmol L^−1^ Fe(CN)_6_^−3^/Fe(CN)_6_^−4^ in 0.1 mol L^−1^ PBS (pH = 7).

Using the cyclic voltammetry (CV) technique, the electrochemical behavior of unmodified (GC/AuNPs) and modified (GC/AuNPs/PMS) electrodes were compared (Figure 3A). As can be seen from Figure 3A, the modified electrodes with GC/AuNPs/PMS affect the current and the reversibility of the redox probe. For the modified electrodes, the current is changed in series: AuNPs/PMS2 (curve green) < AuNPs/PMS1 (curve red) < AuNPs (curve black). This suggests that the electron transfer to the electrode was slowed by the addition of the anion-exchanger receptor. For GC/AuNPs/PMS2, the observed decreasing redox peaks can be attributed to the increasing negative charge in the PVC-plasticized membrane. Leaching of PMS2 was observed from the electrode surface. Therefore, the PMS1 salt was selected as an optimal modifier of the electrode surface and was used in the following experiments.

Electrochemical Impedance Spectroscopy (EIS) was used to check the ionic conductivity of the AuNPs/MS1-modified electrode surface in solutions of mono-(NaCl, NaI), di-(Na_2_SO_4_) and multi-(heparin) charged anionic species (Figure 3B). Moreover, EIS is an additional approach used to evaluate the binding process on the phase boundary through registrating the modified electrode surface resistance. Because it was not possible to fit a semicircle at higher frequencies, the evaluation of the EIS spectra was only performed for the second semicircle observed in the region of lower frequencies (Figure 3B). Parameters of fitting are presented in Table 1. The resistance of the AuNPs/PMS1-modified GC electrode is increased in the following sequence: heparin (1275 Ω) < Na_2_SO_4_ (1487 Ω) < NaCl (1773 Ω) < NaI (7583 Ω). This is because the resistance of the modified electrode surface is affected by the release of iodide. When the specific interaction between the PMS and anionic analytes occurs, the iodide diffuses and causes a decrease in resistance values [28].

### 3.3. Electrochemical Behaviour of Heparin on the Modified Electrode Surface

**Figure 4:**
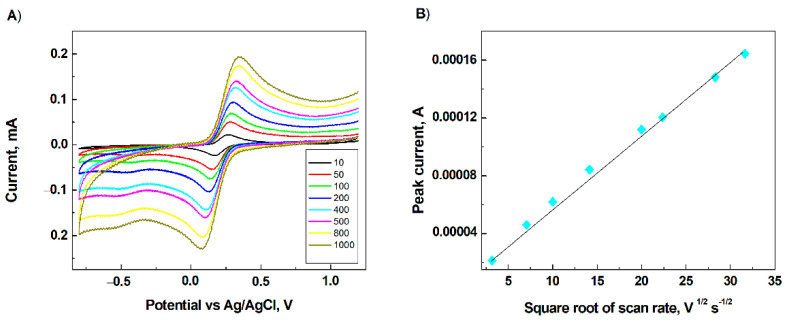
(**A**) Cyclic voltammetric response of the GC/AuNPs/PMS1-modified electrode at scan rates (inner to outer) of 10, 50, 100, 200, 400, 500, 800, 1000 mV s^−1^. (**B**) Peak current dependence on the square root of the scan rate. Supporting electrolyte: 1 mmol L^−1^ Fe(CN)_6_^−3^/Fe(CN)_6_^−4^ in 0.1 mol L^−1^ PBS (pH = 7).

A common method for differentiating between surface-bound and diffusional systems is based on the analysis of the dependence of the peak current on the scan rate (Figure 4A). For PMS1, experimental plots of log|ip| vs. log|ν| for all tested anionic species yielded gradients close to 0.5 (0.44–0.50), which corresponds to a purely diffusional system [29]. Additionally, these results show that the voltammetric response follows the Randles–Ševčík equation (semi-infinite linear diffusion regime) and that the porosity or PVC-plasticized membrane layer effects do not dominate the voltammetric signal under the experimental conditions.

It is possible to propose that the polyanionic analyte, heparin, adsorbed by the PVC-plasticized membrane layer covering the AuNPs/PMS1 on the GC electrode surface, gives access to the inner electroactive surface for an electrochemical reaction for Fe(CN)_6_^−3^/Fe(CN)_6_^−4^ (Figure 4B).

### 3.4. Detection of Heparin on the Modified Electrode Surface

**Figure 5:**
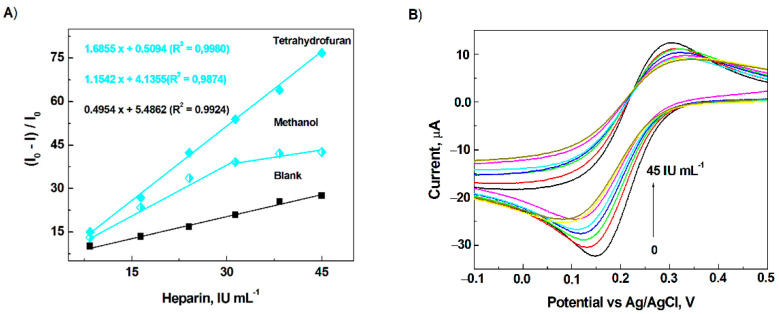
(**A**) Effect of organic solvent used for dispersing of composite AuNPs and PMS1 on the voltammetric response. (**B**) Cyclic voltammograms obtained with the AuNPs/MS1-modified GC electrode after addition of different concentrations of heparin. (Supporting electrolyte: 1 mmol L^−1^ Fe(CN)_6_^−3^/Fe(CN)_6_^−4^ in 0.1 mol L^−1^ PBS (pH = 7). Scan rate 50 mV s^−1^.).

The main purpose was to demonstrate that nanocomposites, including gold nanoparticles and pentamethinium salts covered by a PVC-membrane and deposited on a GC electrode surface, can be used to detect heparin. The PBS buffer is known as Phosphate-Buffered Saline, which is commonly used in biological research. The response of the GC/AuNPs/PMS-modified electrode was measured in PBS as a model system. CV voltammograms were measured on the modified GC electrodes when the composite mixture AuNPs/PMS1 dispersed either methanol or tetrahydrofuran and deposited on the surface of the GC electrode, after being covered with the PVC-plasticized membrane (Figure 5). It should be noted that Meyerhoff et al. [18] reported the first detection of heparin by using a PVC layer. Therefore, we can suppose that the proposed method can be suitable for sensing heparin in a biological matrix. The comparison of the voltammetric response of un-modified and modified electrode surfaces showed that *I*) the sensitivity of the modified electrode towards heparin is higher than unmodified ones and *II*) the solvent used for the composite mixture of AuNPs/PMS1 affects wthe orking concentration range and sensitivity; the least polar solvent (THF) is more favorable than more polar ones (methanol) (Figure 5A). However, only the electrode modified with AuNPs/PMS1 dispersed in THF can be used for the voltammetric determination of heparin in the field of a higher concentration range (31.37–45.02 IU mL^−1^). Tetrahydrofuran is a solvent that is traditionally used for the preparation of plasticized poly(vinyl chloride) (PVC) membranes [18]. One of the advantages of tetrahydrofuran is its ability to rapidly evaporate after deposition on the electrode surface. As demonstrated by Meyerhoff et al. [18,30], there is no issue with using a plasticized PVC membrane prepared using THF for biological applications. It is probable that the solvent of the composite mixture AuNPs/MS1, namely THF—that is compatible with the membrane cocktail PVC-plasticized matrix—plays an important role. The proposed GC/AuNPs/PMS1-modified electrode demonstrated the repeatability signal in the concentration range of 8.41–45.02 IU mL^−1^ 3–5% for 7 days. Signal reproducibility was attained at 12–16% in the working concentration range for three electrode replicates prepared using the same procedure.

The CV measurements of the AuNPs/PMS1-modified GC electrode showed a diffusive voltammetry signal. However, it should be noted that the oxidative and reductive peak heights were not symmetrical (Figure 5B). The changes observed for the oxidative peak were less in comparison with the ones for the reductive peak. It can be concluded that the anionic species, in particular chloride from PBS, having affinity to the PVC-plasticized matrix (Figure 5B), hinder the access of redox markers bearing negative charge to the modified electrode surface and affect the voltammetric signal when scanning in the anodic direction [31].

### 3.5. Analytical Application

Recently, there has been some effort to develop a heparin biosensor. Biosensors have the advantage of not requiring additional reagents nor specific laboratory-based equipment and therefore have potential as a ‘bedside tool’. Ma and co-workers established an ion-selective electrode which uses TDMAC (tridodecylmethylammonium chloride) as an affinity ligand immobilized in a polymer membrane [32]. The concentration range of the proposed sensor was 1.0 to 9.8 IU mL^−1^, which corresponds to the heparin concentration monitored during cardiovascular surgery (where doses between 2 and 8 IU mL^−1^ are commonly used) [32,33]. The concentration of heparin was measured in saline and diluted human serum samples (Table 2). The peak height change observed for the redox marker on the CV voltammograms with the AuNPs/PMS1-modified electrode after heparin addition was used to assess its concentration in the unknown samples. Clearly, the results are in satisfactory agreement with the labeled amounts. As summarized in Table 2, good recoveries were achieved for all concentrations of heparin spiked into the model and biological samples. In the case of model samples, the recoveries for the three concentrations were between 95.1 and 98.5%. In the case of biological samples, the recoveries for the two concentrations were between 99.7 and 100.9%. Taking into account these results, it can be concluded that the proposed sensor can be suitable for the determination of heparin in both saline and the biological system.

## 4. Conclusions

A voltammetric sensor based on nanocomposites including gold nanoparticles and pentamethinium salts dispersed in organic solvent is proposed. To preserve the nanocomposites on the electrode surface, PVC-plasticized membrane was used. The applicability of the proposed sensor concept was checked for heparin detection. For detection, limit lowering and extension of the linear response range, it was necessary to apply a solvent compatible with PVC-plasticized membrane, namely tetrahydrofuran. The component of the biological matrix does not affect the response of the proposed voltammetric sensor.

## Figures and Tables

**Table 1:** Electrochemical parameters of a GC/AuNPs/PMS1 electrode.

EIS–Parameters	Anionic Species			
	Sulfate	Chloride	Iodide	Heparin
*C*_dl_, μF	12.7	16.7	21.1	15.4
*R*_ct_, kΩ	1.48	1.773	7.583	1.275

**Table 2:** Determination of heparin in model samples using the AuNPs/PMS1-modified GC electrode by standard addition method (n ≤ 8).

Sample	Introduced, IU mL^−1^	Found, IU mL^−1^	Recovery, %
Saline	1.72	1.66 ± 0.20	96.5
	8.42	8.29 ± 0.98	98.5
	16.43	15.63 ± 1.09	95.1
Human serum	1.93	1.93 ± 0.37	99.7
	7.50	7.57 ± 0.86	100.9

## Data Availability

The data underlying this article will be shared on reasonable request from the corresponding author.

## Supplementary Materials

The following are available online at https://www.mdpi.com/article/10.3390/ma14185357/s1, Figure S1: the SEM images of the layer of NPOE-plasticized membrane coated electrode surface. The micrographs of the surfaces were taken with a scanning electron microscope JSM 6400 (JEOL, Japan).
