# Supplementary material for: Pentamethinium Salts Nanocomposite for Electrochemical Detection of Heparin
Source: Materials (Basel). 2021 Sep 17;14(18):5357. doi: 10.3390/ma14185357 (PMC8465147; doi:10.3390/ma14185357)
Supplement: Supplementary file 1 [file materials-14-05357-s001.zip › materials-1359094-supp-update.pdf]

materials

MDPI

Supplementary Materials

Pentamethinium Salts Nanocomposite for Electrochemical Detection of Heparin

Tatiana V. Shishkanova [1,2,*], Tomáš Bříza [2,3], Pavel Řezanka [1], Zdeněk Kejík [2,3] and Milan Jakubek [1,2,3]

[1] Department of Analytical Chemistry, University of Chemistry and Technology, Prague, Technická 5, 166 28 Prague 6, Czech Republic; pavel.rezanka@vscht.cz (P.Ř.); milan.jakubek@vscht.cz (M.J.)
[2] BIOCEV, First Faculty of Medicine, Charles University, Průmyslová 595, 252 50 Vestec, Czech Republic; tomas.briza@lf1.cuni.cz (T.B.); zdenek.kejik@lf1.cuni.cz (Z.K.)
[3] Department of Paediatrics and Inherited Metabolic Disorders, First Faculty of Medicine, Charles University and General University Hospital, Kateřinská 1660/32, 121 08 Prague 2, Czech Republic
* Correspondence: tatiana.shishkanova@vscht.cz

Supplementary Materials

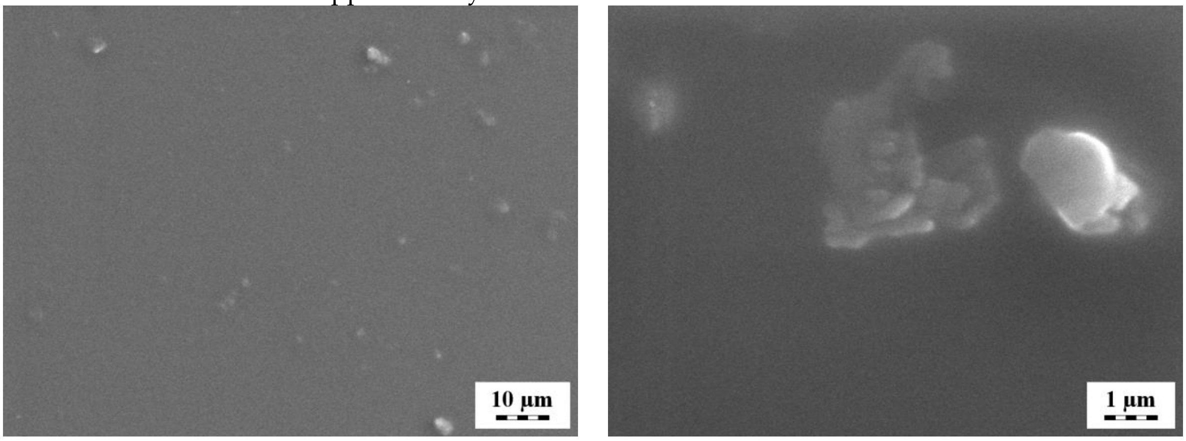

(A) (**B**)

Figure S1. The SEM images of the layer of NPOE-plasticized membrane coating the electrode surface. The micrographs of the surfaces were taken with a scanning electron microscope JSM 6400 (JEOL, Japan).
